# Medical Defense Union

**Published:** 1900-07

**Authors:** 


### MEDICAL DEFENSE UNION.

ATTENTION has been called in these columns to the desirability of establishing a medical defense union for the protection and aid of physicians when they are in position of defendants in lawsuits upon matters touching their profession. Unions of this character have proved very effective in England, and are shown to act as powerful deterrents against that species of legal blackmail of which physcians are so often made the victims. A late issue of the *Northwestern Lancet* contains the intelligence that a movement has been set on foot by the members of the Ramsey County Medical Society to establish a defense union on the English model. A committee was appointed to investigate the system and report the result of the inquiry at the next meeting of the State (Minnesota) Medical Society. This movement will be watched with interest, and it is to be hoped that the example of the Minnesota body in seeking organized protection will be emulated, and be the means of arousing the profession from the supine lethargy which characterizes it in respect of its best interests.

---

A VERY novel experiment to determine whether or no mosquitoes are the cause of malarial infection is now being conducted in Rome, Italy, by two English physicians, Drs. L. W. Sambon and G. C. Low. The two physicians are earnest advocates of the theory that malaria is contracted only through contact with mosquitoes. These two physicians have had a mosquito-proof hut made and placed in the Roman Campagna—long known as the habitat of the most virulent forms of malaria—and they propose to live in this from June to October. On their theory they should not contract malaria, since they will effectually keep away from mosquitoes. We shall await with interest the result of this experiment.

